# Diverse activity of miR-150 in Tumor development: shedding light on the potential mechanisms

**DOI:** 10.1186/s12935-023-03105-3

**Published:** 2023-11-03

**Authors:** Ali Ameri, Hani Moslem Ahmed, Renzon Daniel Cosme Pecho, Hesamoddin Arabnozari, Hoda Sarabadani, Romina Esbati, Seyedsaber Mirabdali, Omid Yazdani

**Affiliations:** 1 Student Research Committee, Faculty of Pharmacy, Hormozgan University of Medical Sciences, Bandar Abbas, Iran; 2 Department of Pharmacy, Al-Noor University College, Nineveh, Iraq; 3 Department of Biochemistry, Universidad San Ignacio De Loyola (USIL), Lima, Peru; 4 School of Medicine, Babol University of Medical Sciences, Babol, Iran; 5 Rajiv Gandhi Institute of Information Technology & Biotechnology, Bharati Vidyapeeth University, Pune, India; 6 Department of Medicine, Shahid Beheshti University, Tehran, Iran; 7 Regenerative Medicine Research Center, Kermanshah University of Medical Sciences, Kermanshah, Iran

**Keywords:** MiR-150, Cancer, Metastasis, Migration, Invasion

## Abstract

There is a growing interest to understand the role and mechanism of action of microRNAs (miRNAs) in cancer. The miRNAs are defined as short non-coding RNAs (18-22nt) that regulate fundamental cellular processes through mRNA targeting in multicellular organisms. The miR-150 is one of the miRNAs that have a crucial role during tumor cell progression and metastasis. Based on accumulated evidence, miR-150 acts as a double-edged sword in malignant cells, leading to either tumor-suppressive or oncogenic function. An overview of miR-150 function and interactions with regulatory and signaling pathways helps to elucidate these inconsistent effects in metastatic cells. Aberrant levels of miR-150 are detectable in metastatic cells that are closely related to cancer cell migration, invasion, and angiogenesis. The ability of miR-150 in regulating of epithelial-mesenchymal transition (EMT) process, a critical stage in tumor cell migration and metastasis, has been highlighted. Depending on the cancer cells type and gene expression profile, levels of miR-150 and potential target genes in the fundamental cellular process can be different. Interaction between miR-150 and other non-coding RNAs, such as long non-coding RNAs and circular RNAs, can have a profound effect on the behavior of metastatic cells. MiR-150 plays a significant role in cancer metastasis and may be a potential therapeutic target for preventing or treating metastatic cancer.

## Introduction

Metastasis is known as the spread of malignant cells from the main tumor to some other area of the body through blood and lymph vessels, as well as their colonization and proliferation in the new region. This process is generally considered inefficient and only those survivors may spread to target organs under ideal circumstances [1]. Primary tumor cells are normally exposed to a wide range of environmental pressures, such as low pH, deficiency in oxygen, increased reactive oxygen species, and lack of nutrients [2]. These stressors may induce cancer cells to adopt an aggressive nature and phenotype. According to cellular and molecular insight, a primary tumor is composed of various cell clones with different properties, and rare clones acquire the ability to metastasize. Genomic and chromosomal instability increases certain mutations and alterations that cause tumor heterogeneity and the development of cancer cells with migration and invasion capacity [3]. Besides, abnormal cell cycle, impairment of DNA repair mechanism, telomere crisis, and epigenetic changes may affect DNA integrity and tumor heterogeneity [4]. Furthermore, various signaling pathways, a broad spectrum of subcellular functions, and regulatory apparatuses, such as microRNAs (miRNAs), are involved during tumor metastasis to facilitate cancer cell invasion.

MiRNAs are defined as evolutionarily conserved non-coding RNA (18–22 nucleotide) that mainly interact with 3′ UTR of mRNA. This class of short non-coding RNAs post-transcriptionally modulates mRNA, which inhibits the translation of target mRNA or causes mRNA degradation. In 1993, LIN-4 was the first miRNA reported to inhibit LIN-14 in C. elegans [5]. Nowadays, over 2,600 mature miRNAs have since been discovered, and it is believed that they are responsible for controlling more than half of the protein-coding genes in humans [6]. The genes of miRNA are found as individual genes or within introns of protein-coding genes. RNA polymerase II mainly transcribes related genes as primary miRNA that consists of a 5′ cap, 3′ poly(A) tail, and hairpin structure [7]. The primary miRNAs are processed into functional or mature miRNAs during the biogenesis process. The levels of miRNAs are strictly controlled in many biological pathways and are essential for typical mammalian development. Therefore, many human disorders such as cancer can be linked to abnormal miRNA expression.

The miR-150 has been extensively explored in both normal physiology and various kinds of cancer. MiRNA expression analysis revealed that miR-150 is among the most down-regulated miRNAs in different malignancies including liver, ovarian, pancreatic, colorectal, and neck squamous cell carcinoma [8–11]. It is speculated that miR-150 acts as a tumor suppressor gene in these mentioned cancers. On the other hand, overexpression of miR-150 in breast cancer has been documented. Due to stimulating tumor development and suppressing cell death, miR-150 is an oncogene in this type of cancer [12]. Interestingly, the evaluation of 165 triple-negative breast cancer and 59 control specimens has indicated that miR-150 is down-regulated in tumor specimens and has tumor suppressor activity [13]. As a consequence, the inconsistent effect of miR-150 is found not only in different cancers but also in tumors of the same cancer type.

In this comprehensive review, we focused on miR-150 levels and potential targets in non-cancerous cells during migration to provide wide insight into the biological function of miR-150. The regulation of the EMT process via miR-150 by emphasizing EMT-related transcription factors (TFs) and signaling pathways was reviewed. MiR-150 can regulate the migration of cancer cells by affecting migration-associated matrix metalloproteinases (MMPs), cell adhesion molecules, epigenetics modulators, and TFs. Tumor microenvironment factors and tumor-associated cells such as macrophages modulate angiogenesis through miR-150.

## MiR-150 biological function in migration of non-cancerous cells and related Diseases

Over the past decade, numerous investigations have described the miR-150 as a key regulator of biological processes such as differentiation, apoptosis, proliferation, and autophagy. As shown in Table 1, miR-150 has dynamic expression levels in different cells, acting as a member of regulatory networks and changing cell migratory behavior. Depending on cell content, a plethora of genes and signaling pathways have been considered as the miR-150 targets. Identification of these sophisticated regulatory pathways and potential targets of miR-150 in biological processes give rise to a better understanding of the pathology of diseases such as cancer.

**Table 1:** miR-150-mediated modulation of migration in non-cancerous cells and related diseases

Cell type	Species	Exp.	Target genes	Description of target gene	Mig.	Potential diseases	Ref
Bone marrow-derived endothelial progenitor cells	Rat	↓	c-Myb	Inhibiting migration and tube formation of endothelial cells	↓	Deep venous thrombosis	[110]
Brain microvascular endothelial cells	Mouse	↑	Vezf1	A zinc finger transcription factor involved in tube formation and angiogenesis	↓	Fetal alcohol spectrum disorders	[111]
Human aortic smooth muscle cells and mice model	Human and Mouse	↓	STAT1	A transcription factor which is mainly increases cell motility	↑	Atherosclerosis	[112]
HTR-8/SVneo (a trophoblast cell line) and placenta cells	Human	↑	VEGF and MMP9	VEGF and MMP9 contribute to extravillous trophoblast cell migration to induce placental vascular remodeling during pregnancy	↓	Preeclampsia	[113]
Isolated monocytes from human and mouse model	Human and Mouse	↓	CXCR4	The CXCR4 receptor binds its ligands and triggers cell migration	↑	Acute myocardial infarction	[114]
Pulmonary artery smooth muscle cells	Human	↓	HIF-1a	HIF-1α has been shown to elevate cell motility through the activation of RhoA	↑	Pulmonary arterial hypertension	[115]
Brain microvascular endothelial cells and rat model	Human and Rat	↑	VEGF	It is involved in angiogenesis during poststroke recovery	↓	Poststroke recovery	[116]
CRL-7566 cells (derived from an ovarian cyst wall from a patient)	Human	↑	PDCD4	A tumor suppressor protein that is involved in EMT suppression	↑	Endometriosis	[117]
Bone marrow-derived mononuclear cells	Human	↑	CXCR4	The receptor of stromal cell-derived factor that has an essential role in stem cell migration	↓	Ischemia	[118]
Mouse model of oxygen-induced proliferative retinopathy	Mouse	↑	CXCR4, DLL4, and FZD4	miR-150 targets angiogenesis-related genes and reduce endothelial cell migration and tubular formation	↓	Pathologic ocular neovascularization	[119]
Leiomyoma cells	Human	↓	Akt	Akt is a crucial cell survival and migration regulator.	↑	Uterine leiomyoma	[120]

## Epithelial-mesenchymal transition and miR-150

Through a process known as epithelial-mesenchymal transition (EMT), epithelial cells lose their epithelial characteristics and obtain mesenchymal cells phenotype [14]. A wide range of factors such as microenvironmental stimuli causes tumor cells to initiate the EMT process. In the epithelial state, tumor cells are defined by apical–basal polarity, cell–cell junctions, and cell integration to the basement membrane. Due to changes in the post-translational regulatory process and gene expression, these epithelial features are suppressed and tumor cells acquire a fibroblast-like morphology [14]. In addition, cellular composition changes EMT and mesenchymal-like tumor cells have vimentin-based intermediate filaments and bind to the extracellular matrix through focal adhesions expressing integrin. MiR-150 is involved in EMT by regulating related TFs and signaling pathways (Table 2).

**Table 2:** miR-150 is known as a modulator of epithelial-mesenchymal transition in various cancer

Type of cancer	Expression	Non-coding RNA	Target gene	Description of target gene	Signaling pathway	Ref
Ovarian and esophageal squamous cell carcinoma	Decreased		ZEB1	EMT-associated transcription factor		[18, 19]
Non-small cell lung cancer	Decreased	Linc00673				[21]
Osteosarcoma	Decreased	MIAT				[22]
Oral squamous cell carcinoma	Decreased		HMGA2	This transcription factor modulates several genes involved in EMT		[24]
Non-small cell lung cancer	Increased		FOXO4	FOXO4 is known as NF-κB/Snail axis inhibitor	NF-κB	[26]
Ovarian	Increased		c-Myb	Inhibiting c-Myb leads to the induction of Slug levels, an EMT-associated transcription factor		[29]
Colorectal cancer	Increased		EP300 and CREB1	Transcription factors related to CREB signaling pathway	Wnt/β-catenin and CREB	[33]
Prostate cancer	Decreased		TRPM4	This non-specific ion channel promotes the activation of the Wnt/β-catenin signaling pathway	Wnt/β-catenin	[37]
Cervical carcinoma	Increased		SRCIN1	A tumor suppressor	SRC tyrosine kinase	[40]
Lung cancer	Increased					[42]
Breast cancer	Increased					[43]
Gastric cancer	Increased					[44]
Hepatocellular carcinoma	Decreased		GAB1	a scaffolding linker that regulates signal transmission between receptors and subsequent signaling pathways	ERK	[9]
Melanoma	Decreased	circVANGL1			TGFβ	[50]

### Transcription factors

EMT is controlled by a limited set of transcription factor families, including the Zinc finger (such as Snail and ZEB), and basic helix-loop-helix (such as Twist) [15]. MiRNAs and EMT- related TFs form a complex interactome that is capable of sensing various signals from the microenvironment and relaying them to gene expression. Although research has been conducted on the interaction between miR-150 and EMT-related zinc finger transcription factors, there is no single study investigating miR-150 and basic helix-loop-helix TFs interaction in EMT.

#### Direct regulation of zinc finger TFs via miR-150

From yeast to humans, zinc finger TFs are evolutionarily conserved and have a zinc finger binding domain to interact with DNA and other targets [16]. Zinc finger domains rely on the presence of a zinc ion coupled with two cysteine and two histidine residues for their function and structure [17]. Slug, Snail, and ZEB are members of zinc finger TFs that regulate EMT-related biological markers.

The transcription of miR-150 in esophageal squamous cell carcinoma and ovarian cancer is lower than in healthy specimens, according to a microarray database and experimental methods [18, 19]. A novel miR-150 target, ZEB1, is determined. ZEB1 has been historically linked to the development of cancer and is required for EMT [20]. Targeting ZEB1 with miR-150 can increase E-cadherin expression (an epithelial cell marker) and inhibits esophageal squamous cell carcinoma and ovarian cancer development [18, 19]. One of the reasons for the reduction of miR-150 expression can be long non-coding RNA (lncRNAs), which has been proven in non-small cell lung cancer and osteosarcoma [21, 22]. It is recognized that the interaction between miRNA and lncRNAs is essential for gene regulation. LncRNAs perform as sponges that competitively bind to desired miRNAs and decrease their effects on associated mRNA [23]. Linc00673 and MIAT have a complementary binding site for miR-150 and reduce its function by sponging in non-small cell lung cancer and osteosarcoma, respectively. Therefore, ZEB1 levels are indirectly affected and EMT is promoted in both types of cancer [21, 22].

#### miR-150 indirectly modulates zinc finger TFs

The regulation of EMT at the molecular level through miR-150 and other less relevant TFs has been reported. It seems that miR-150 acts as a tumor suppressor in oral squamous cell carcinoma and its down-regulation is associated with poor prognosis and metastasis [24]. In light of further analysis, HMGA2 (the high mobility group A2) transcription factor is determined as a miR-150 direct target. This transcription factor modulates several genes involved in EMT including E-cadherin, N-cadherin, and snail [25]. Overexpression of miR-150 reduces EMT in oral squamous cell carcinoma by inhibiting HMGA2. In contrast, miR-150 acts as an oncogene and promotes EMT in human non-small cell lung cancer [26]. In metastatic non-small cell lung cancer specimens and cell lines, miR-150 levels are significantly increased and target FOXO4 (Forkhead box protein O4). Based on the promoter’s region and extracellular circumstances, this transcription factor either represses or activates gene expression [27]. FOXO4 low-level expression is found in non-small cell lung cancer and may contribute to EMT [28]. It has been shown that FOXO4 is known as NF-κB/Snail axis inhibitor and its downregulation provides the high activity of the NF-κB/Snail axis [26]. Eventually, EMT-inducing Snail leads to E-cadherin inhibition, vimentin induction, and N-cadherin up-regulation. Slug, a member of the Snail transcription factor family, is indirectly regulated via miR-150. Compared to primary tumors, miR-150 is elevated in recurrent tumors and enhances cell migration and EMT in ovarian cancer cells [29]. Indeed, miR-150 causes EMT via upregulating Slug by inhibiting c-Myb. The results of this investigation were somewhat unexpected. Slug expression is generally activated by c-Myb [30, 31]; however, in this investigation, c-Myb suppresses Slug in ovarian cancer tissue. It seems that the c-Myb function may be associated with cell type.

### Signaling pathway

To induce EMT, various signaling pathways and molecules trigger corresponding receptors on the cell surface, which in turn activates a downstream signaling cascade that ultimately activates EMT TFs and their related co-regulators and epigenetic moderators [32]. Interestingly, similar to transcription factors, a particular miRNA may directly regulate the expression of dozens of genes by influencing cell signaling pathways. The question that arises is how miR-150 exerts its effects on EMT via associated signaling pathways.

#### Wnt/β-catenin

**Fig. 1:**
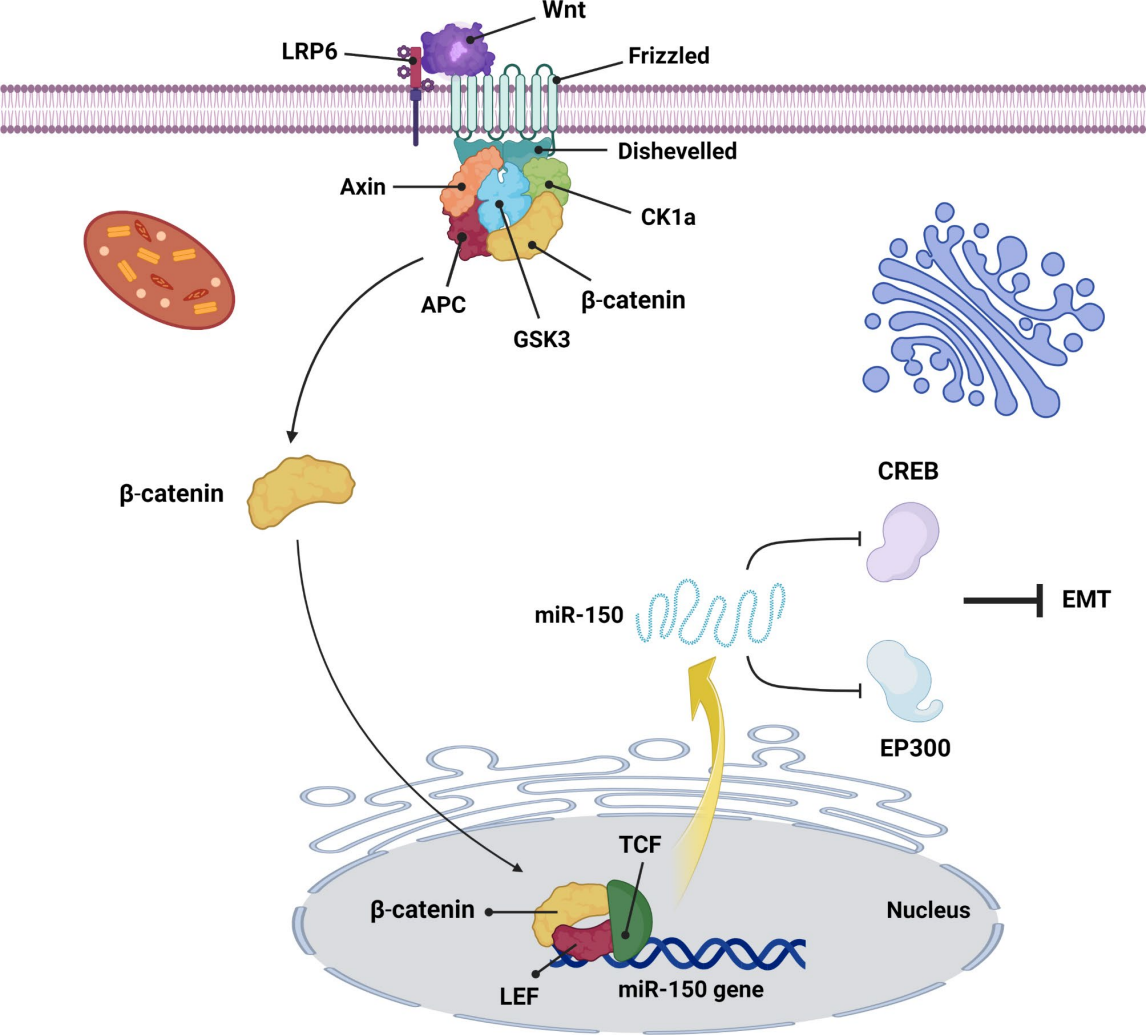
The miR-150/CREB axis in cancer. The miR-150 inhibits the CREB signaling. MiR-150 inhibits the CREB signaling pathway and thus affects EMT process

Guo et al. have described a molecular mechanism in which the Wnt/β-catenin and CREB signaling pathways are up and downstream of miR-150 in colorectal cancer, respectively [33]. Indeed, miR-150 has a central role in the interaction of two different types of signaling pathways. Upregulation of Wnt/β-catenin triggers miR-150 expression by binding the β-catenin/LEF1 component in the promotor region. As a result, overexpressed miR-150 significantly inhibited the CREB signaling pathway via binding to EP300 and CREB1 transcription factors (Fig. 1). Down-regulation of the CREB signaling pathway leads to EMT in colon cancer. However, CREB is mentioned as an oncogenic transcription factor in some cancer [34], the results of the investigation indicated that this transcription factor plays a tumor suppressor role in several cancers and its low-level expression promote metastasis [35]. EP300 is also typically low expressed in various forms of cancer, such as colon cancer and breast cancer [36]. Interestingly, Yu et al. have indicated that miR-150 can indirectly down-regulate the Wnt/β-catenin signaling pathway and suppresses EMT in prostate cancer [37]. According to microarray-based analysis, miR-150 and TRPM4 (transient receptor potential melastatin 4) have been down and up-regulated in prostate cancer tissue. TRPM4 is a non-specific ion channel accessible to K + and Na + that is stimulated via Ca2+ [38]. Current research indicates that TRPM4 promotes the activation of the Wnt/β-catenin and prostate cancer malignancy [39]. MiR-150 binds to TRPM4 mRNA and represses its biological function. Consequently, the down-regulation of TRPM4 results in reducing the Wnt/β-catenin signaling pathway and EMT [37].

#### SRC tyrosine kinase

In cervical carcinoma cells, miR-150 serves as an oncogene in a different manner [40]. miR-150 has been found high-level expression in C-33 A and HeLa cervical carcinoma cell lines and promotes EMT features [40]. The analysis confirmed that miR-150 facilitates tumor malignancy by targeting a tumor suppressor, SRCIN1 (SRC kinase signaling inhibitor 1). Src signaling pathways, which are cellular tyrosine kinases, are typically overexpressed or abnormally activated in cancer cells [41]. As an effective tumor suppressor, SRCIN1 can suppress Src signaling pathways and downstream epidermal growth factor receptors, and focal adhesion kinases. Moreover, SRCIN1 was explored in lung, breast, and gastric cancer cells and its relation with miR-150 has been shown [42–44]. Overexpressed miR-150 causes A540 lung cancer cells, breast cancer cell lines, and BGC-823 gastric cancer cells to acquire malignancy behavior through suppressing SRCIN1.

#### ERK signaling pathway

The miR-150 has tumor suppressor activity in hepatocellular carcinoma. According to hepatocellular carcinoma tissue evaluation, miR-150 is down-regulated upon metastasis [9]. On the other hand, GAB1 (Grb2-associated binding protein 1) has high-level expression in hepatocellular carcinoma tissue and is determined as a miR-150 target. The role of GAB1 in promoting EMT is not a surprise [45]. In fact, GAB1 is a scaffolding linker that regulates signal transmission between receptors and subsequent signaling pathways [46]. GAB1 triggers ERK signaling pathway activation, which is a key coordinator of EMT [47].

#### TGFβ signaling pathway

Transforming growth factor-β (TGFβ) is a cytokine binding to its specific cell surface receptor and triggers a signal from the cell membrane to the nucleus through canonical or non-canonical pathways [48]. Therefore, a wide range of genes undergoes regulation by TGFβ signal transduction, and consequently, various cellular functions such as proliferation, migration, apoptosis, cell polarity, and cytoskeleton restructuring are adjusted. This is perfectly sensible that aberrant activation of the TGFβ signaling pathway is related to human cancers. In the case of the TGFβ signaling pathway, it is accepted that TGFβ has tumor-suppressing actions in the beginning stages of cancer by preventing cell proliferation and promoting programmed cell death; however, TGFβ stimulates tumor metastasis in the advanced stage of the tumor [49]. The involvement of the TGFβ signaling pathway with the miRNA regulatory network might give further explanation for the inconsistent effect of TGFβ in cancer.

**Fig. 2:**
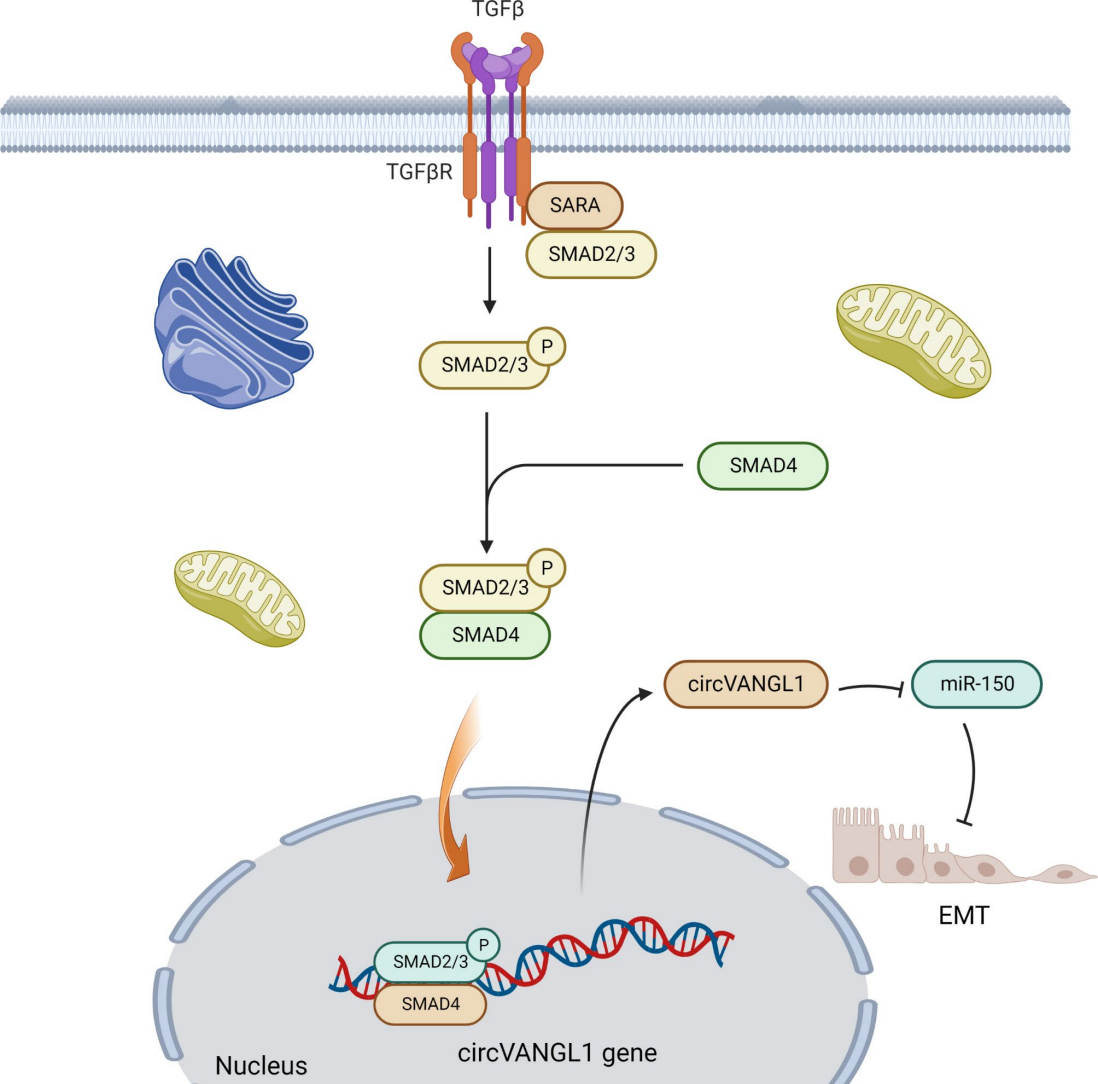
TGFβ/circVANGL1/miR-150 pathway in cancer. TGFβ singling pathway induces circVANGL1 levels and down-regulates miR-150 by sponging

In melanoma cells, circVANGL1(circular RNA VANGL1) is increased in melanoma cell lines and tissues by TGFβ [50]. Circular RNAs are a significant class of lncRNAs that have a single-stranded ring structure without a 3’ Poly A tail and a 5’ cap [51]. The sponging role of circVANGL1 has been observed in melanoma cells. During the EMT process, TGFβ singling pathway induces circVANGL1 levels and down-regulates miR-150 by sponging (Fig. 2) [50]. Down-regulation of miR-150 is related to an advanced stage of melanoma tumors.

## MiR-150 role in the Tumor cells migration

Cancer cell migration is an essential step during metastasis. A broad spectrum of cellular and molecular apparatuses facilitates migratory phenotype in this sophisticated process [52]. The cytoskeleton-based protrusion, cell adhesion, retraction, and contraction are the hallmarks of front-rear polarization during cell migration [53]. According to front-rear polarization, intracellular proteins trafficking, and sorting have a pivotal role in the distribution of cell adhesion proteins such as integrin on the cell membrane. This enables the migrating cells to properly interact with the extracellular matrix and microenvironmental components that promote tumor cell invasion [54]. Different regulations of migration-associated genes via miR-150 lead to the commitment of cancer cells to migration and invasion (Table 3).

**Table 3:** Direct targets of miR-150 and Its outcomes in migration of various cancer cells

Biological component	Cancer	Up or Down-regulation	Direct target	Description	Ref
Matrix metalloproteinases	Cervical	Up	PDCD4	PDCD4 is an inhibitor of MMP-9. Targeting PDCD4 elevates MMP-9 levels.	[57]
	Glioblastoma	Down	MT1-MMP	MiR-150 targets MT1-MMP, a type of MMPs family	[58]
	Hepatocellular	Down	MMP14	MMP14 degrades an ECM and facilitates cancer cells migration	[60]
	lung	Down	MMP14	FAM83A-AS1 is a lncRNA that down-regulates miR-150 directly and increases MMP14 levels indirectly.	[61]
	Breast	Down	MMP13	miR-150 is down-regulated by LINC00511 and MMP13 is upregulated	[62]
Cell adhesion molecules	Prostate and head and neck squamous cell	Down	SPOCK1	Ca2+-binding proteoglycan, SPOCK1 has a role in cell-cell and cell-matrix interaction	[63, 64]
	Head and neck squamous cell	Down	ITGA3, ITGA6 and TNC	Integrins and TNC are transmembrane receptors and ECM-related glycoproteins, respectively.	[11]
	Colorectal and pancreatic	Down	MUS4	MUS4 is a transmembrane glycoprotein that is enhanced in cancers and promotes metastasis	[70, 71]
Transcription factors	Melanoma	Down	MYB	MYB encodes a migration-mediated transcription factor	[75]
	Colorectal	Down	iASPP	iASPP is the inhibitor of the p53 transcription factor	[76]
	Ovarian	Down	Sp1 and ZFAS1	miR-150-5p is inhibited by ZFAS1, and Sp1 is upregulated and triggers the expression of migration-related genes.	[81]
	Ovarian	Down	MIAT	MIAT is lncRNA sponging miR-150	[82]
Epigenetics	Breast	Down	HMGA2	Up-regulation of HMGA2, a chromatin remodeling-related factor, triggers breast cancer cell migration.	[84]
	Thyroid	Down	EZH2	Upregulation of MIAT sponges miR-150 and induces EZH2 levels, which is important for thyroid cancer cell migration.	[86]

### Extracellular matrix

The tumor microenvironment is a dynamic system that determines tumor progression and metastasis through interaction between involving cells and extracellular matrix (ECM) macromolecules. It is currently accepted that the extracellular matrix is a crucial regulator of numerous cell biological activities, including tissue remodeling. The ECM changes due to a variety of remodeling mechanisms, which can be broadly categorized into four distinct processes [55]: (1) post-translational chemical alteration that modifies the ECM’s molecular properties and structural features; (2) ECM deposition modifies the quantity and content of ECM mechanical and biochemical factors; (3) Physical restructuring induced by force, which affects ECM architecture through orienting ECM filaments and providing pathways for cell migration; (4) Proteolytic degradation refers to the release of proteolytic enzymes such as matrix metallopeptidase (MMPs) that degrade matrix proteins. There is broad agreement that miRNAs play a crucial role in metastasis due to the abundance of data linking ECM modification during migration and invasion to miRNAs [56].

#### Matrix metalloproteinases

MiR-150 is elevated in specimens of cervical cancer and leads to cell migration and invasion by inhibiting PDCD4 [57]. PDCD4 decreases MMP-9 levels through negative regulation of the PI3K pathway. In fact, PDCD4 is regarded as the upstream inhibitor of MMP-9, a modulator of the extracellular matrix via decomposing of collagenous substrates. Other members of the MMP family may be targeted through miR-150 directly. MiR-150 binds to MT1-MMP mRNA and inhibits its expression in glioblastoma [58]. MT1-MMP is a transmembrane membrane proteinase type 1 that belongs to the MMP family. This MMP has been recognized as a key component in cancer progression [59]. Due to the down-regulation of miR-150, MT1-MMP is increased and tumor cell migration is promoted. Furthermore, Zhu et al. have illustrated that the induction of miR-150 levels in hepatocellular carcinoma cells results in reducing the development of hepatocellular carcinoma by inhibiting the migration of malignant carcinoma cells via targeting MMP14 [60]. Furthermore, the miR-150/ MMP14 regulation pathway can be modulated by lncRNAs during tumor cell migration. The enhanced levels of FAM83A-AS1 increase MMP14 expression and lung adenocarcinoma cell migration through sponging miR-150 [61]. LINC00511 is another lncRNA that is highly expressed in breast cancer, and its elevated levels are associated with tumor malignancy in breast cancer patients [62]. Subsequent analysis has shown that elevated LINC00511 levels are accompanied by high-level expression of MMP13. MiR-150 is inhibited by LINC00511 and its direct target (MMP13) is up-regulated.

#### Cell adhesion molecules

There is evidence of the ability of miR-150 to direct the targeting of extracellular matrix components. According to an investigation on metastatic prostate cancer and head and neck squamous cell carcinoma specimens, the high-level expression of the SPOCK1 gene and down-regulation of miR-150 has a significant role in the malignant behavior of tumor cells [63, 64]. SPOCK1 is a Ca^2+^-binding proteoglycan that contributes to cell-matrix and cell-cell interactions during migration [65]. Bioinformatic and dual-luciferase assays revealed that miR-150-3p (passenger strand) and miR-150-5p (guide strand) directly target SPOCK1. In miRNA biogenesis, Dicer cleaves pre-miRNA into the miRNA duplex, which contains the guide and passenger strands [66]. The mature guide strand miRNA is integrated into the RISC and represses or cleaves target mRNA. While the passenger strand of miRNA was considered to be destroyed and ineffective [67], miR-150-3p can target SPOCK1. Besides, miR-150-5p and − 3p targets ITGA3 (integrin alpha 3), ITGA6 (integrin alpha 6), and TNC (tenascin C) in head and neck squamous cell carcinoma [11]. TNC is a glycoprotein that is released in the ECM During the process of cancer cell migration [68]. As well, integrins are defined as transmembrane receptors that facilitate cell-matrix adhesion [69]. Decreasing in miR-150 level triggers tumor cell migration via increasing ITGA3, ITGA6, and TNC expression [11].

Investigations are showing that miR-150 binds to MUS4, a cell transmembrane glycoprotein with a high molecular weight [70]. MUC4 is mainly involved in survival-related cell signaling, cell adhesion to ECM, and cell-cell interaction. Its expression is elevated during tumor metastasis causing the detachment of metastatic cells from the primary tumor and impairment in the cell adhesion process, which is required for cancer cell migration. Low-level expression of miR-150 in colorectal [70] and pancreatic [71] cancer has a negative correlation with a high level of MUS4 and tumor metastasis. Up-regulation of miR-150 decreases tumor cell migration by targeting MUS4. Furthermore, it has been illustrated that miR-150 target CLDN12 mRNA, a transmembrane protein, and reduce pancreatic adenocarcinoma cell migration [72]. CLDN12 is a member of the claudins family that is involved in cell-cell interaction, and its high-level expression facilitates the malignant phenotypes of pancreatic adenocarcinoma cells. On the other hand, LINC00857 is a lncRNA that elevates CLDN12 expression via sponging miR-150 [72]. The overexpression of LINC00857 results in adenocarcinoma cell migration and invasion.

### Transcription factors

Gene regulation is a fundamental process in biological mechanisms that can be complex and dynamic [73]. A disruption in the controlling of gene expression may result in inappropriate cell function [74]. MicroRNAs and TFs are two essential kinds of gene expression regulators: miRNAs regulate gene expression at the post-transcriptional level by binding to 3′ untranslated regions of mRNA, whilst TFs regulate gene expression at the level of transcription by engaging to promoter regions. Significantly, miRNAs and TFs can directly or indirectly regulate each other.

The evaluation of the miRNA- mRNA interaction in melanoma specimens revealed that miR-150 can bind the mRNA of the MYB gene and suppresses its expression at the protein level. However, miR-150 downregulation facilitates cancer cell migration by increasing MYB expression [75]. MYB is a proto-oncogene that codes a transcription factor involved in the regulation of cell proliferation, differentiation, and migration. In colorectal cancer clinical samples, miR-150 was downregulated, whereas iASPP (inhibitor of apoptosis-simulating protein of p53) was upregulated [76]. Further research has shown that the migration and invasion of SW480 colon cancer cells are reduced when miR-150 is stimulated [76]. Overexpression of miR-150 and knockdown of iASPP had an identical effect on SW480 cells. Indeed, p53 is the most well-known transcription factor that not only induces cancer cell senescence and apoptosis but also functions as a major suppressor of cancer cell motility and metastasis [77].

The regulation of migration-related TFs through miRNA can be affected by other regulatory elements such as lncRNAs. High-level expression of ZFAS1, a lncRNA, has been observed in several human cancers, such as colorectal cancer, gastric cancer, and hepatocellular carcinoma [78–80]. In ovarian cancer, increased levels of ZFAS1 bind to miR-150-5p and inhibit its normal function. Subsequently, the miR-150-5p downstream target, transcription factor Sp1, is elevated and promotes migration-associated gene expression [81]. lncRNA MIAT (myocardial infarction-associated transcript) is highly expressed in SKOV3 and OVCAR3 ovarian cancer cells [82]. The cell migration and invasion are stimulated by overexpression of MIAT and inhibited by knockdown of MIAT. On the other hand, over-expression of miR-150 leads to the downregulation of ovarian cancer cells. MIAT essentially acts as a sponge for miR-150-5p and modulates its activity [82].

### Epigenetics

There is various evidence for the interaction between miRNA regulatory networks and chromatin remodeling [83]. Chromatin is a complex molecular structure in the eukaryotic cell that contains nucleosomes, the wrapped DNA around histone octamers. DNA methylation, histone modifications, and binding of chromatin-remodeling complexes regulate chromatin features and downstream gene expressions [83]. In turn, chromatin modulators can be regulated by miRNAs in a broad regulation network. It has been shown that miR-150 has a negative correlation with HMGA2, a chromatin remodeling protein, in triple-negative breast cancer [84]. Low-level expression of miR-150 results in high expression of HMGA2 and thus promoting breast cancer cell migration. The mounting investigation has revealed that HMGA2 levels are increased during tumor malignancy, and HIF-1α and VEGF are among the downstream genes that are induced by HMGA2, and trigger tumor cell migration [85]. Epigenetics features can be changed by involving the lncRNA-miR-150 axis during cancer cell migration. High-level expression of MIAT, a lncRNA, is found in thyroid cancer progression that has a positive correlation with EZH2 (enhancer of zest homolog 2) [86]. This histone-lysine N-methyltransferase enzyme is associated with histone methylation and transcriptional inhibition [87]. EZH2 overexpression is often associated with advanced malignant tumor stages and poor prognosis [88]. MiR-150 targets EZH2 and reduce tumor cell migration and invasion. The up-regulation of MIAT sponges miR-150 and causes induction of EZH2 expression and thyroid cancer cell migration [86].

## MiR-150 and cancer cell proliferation and apoptosis

The processes of cell proliferation and apoptosis are fundamental to the development and survival of multicellular organism. The primary function of proliferation is to increase the number of cells in a tissue or organ [89]. It is responsible for tissue growth, tissue repair after injury, and the replacement of old or damaged cells. On the other hand, the main role of apoptosis is to remove damaged or unnecessary cells from a tissue or organ [90]. Therefore, the balance between apoptosis and proliferation is a critical aspect of tissue homeostasis and its dysregulation leads to pathological condition such as cancer.

MiR-150 can regulate cell growth by targeting the cell receptors involved in proliferation and apoptosis. Evaluation of miR-150 level in pancreatic cancer cells showed that its expression is decreased and has a negative correlation with the induction of apoptosis [91]. The miR-150 binds to insulin-like growth factor 1 receptor (IGI-1R), a cell surface receptor involved in cell growth, proliferation, and survival. Up-regulation on this miRNA by using mimic method causes down-regulation of IGI-1R and induction of cell death. In breast cancer cell lines, miR-150 has high expression and trigger cancer cell growth and proliferation [12]. By targeting P2 × 7 receptor, miR-150 exerts its anti-apoptotic effects. P2 × 7 receptor induces cell death via activating caspases 3 and 7 [92].

The interaction between lncRNAs and miR-150 in modulation of cell death and proliferation have been investigated recently. Decreasing NEAT1 expression, a long non-coding RNA, and increasing miR-150 levels have key role in the colorectal cancer cells resistance against apoptosis [93]. Further analysis revealed that NEAT1 sponges miR-150 and results in the upregulation of CPSF4, a direct target of miR-150. CPSF4 is a protein involved in the processing of mRNA precursors during mRNA biogenesis [94]. Overexpression of CPSF4 trigger cell proliferation and inhibit apoptosis with distinct mechanism in several cancer [95–97]. Furthermore, PART1, a previously discovered lncRNA, has been shown to has oncogenic properties in many types of cancer [98, 99]. PART1 was shown to be overexpressed in colorectal cancer cell lines and tissues [100]. Reducing PART1 activity inhibited colorectal cancer cell proliferation and increased apoptosis. When considering the molecular features, PART1 up-regulated CTNNB1 by sponging miR-150-5p, which activated the Wnt/ β-catenin pathway. CTNNB1, also known as β-catenin, is a crucial protein that plays a central role in the Wnt signaling pathway [101]. MAFG‑AS1 is an another lncRNA which has high expression in breast cancer cells and facilities cancer cell proliferation by sponging miR-150 [102]. Down regulation of miR-150 provide MYB overexpression and tumorigenesis. MYB is a transcription factor that plays a vital role in the regulation of gene expression [103].

## MiR-150 role in angiogenesis

Angiogenesis is critical for tumor development and metastasis and is activated by chemical signals from cancer cells and the tumor microenvironment [104]. When tumor cells are actively starved for nutrition and oxygen, angiogenesis is triggered. Inhibitory and activating molecules affect angiogenesis. While increased angiogenic factor activity is necessary for tumor angiogenesis, negative regulators must also be suppressed [105]. More than a hundred proteins such as VEGF (vascular endothelial growth factor), bFGF (basic fibroblast growth factor), and angiogenin have been discovered as angiogenic inducers. By controlling the expression of many different related genes, miRNAs play an essential role in tumor angiogenesis [106].

As mentioned above, the tumor microenvironment has a profound effect on tumor angiogenesis. Following the research of Zhang et al. in tumor-associated macrophages (TAM), miR-150 has been shown to increase VEGF secretion from TAM and promote angiogenesis in mouse cancer models [107]. While VEGF is not a direct target of miR-150, this miRNA may regulate VEGF by focusing on upstream proteins. Based on molecular analysis, it has been shown that ING4 (inhibitor of growth family member 4) is a potential target of miR-150 in TAM. ING4 is an inhibitor for the function of HIF (hypoxia-inducible factor), which plays a crucial role in angiogenesis via inducing VEGF [108]. The pro-angiogenic activity of miR-150 has been confirmed in the tumor-associated monocyte [109]. The release of miR-150 from monocytes induces endothelial cell tube creation in vitro and in vivo assessments, while downregulation of miR-150 in monocytes prevents angiogenesis in breast cancer, non-small cell lung cancer, hepatocellular carcinoma, and colon cancer. However, the underlying mechanism and potential targets of monocyte-derived miR-150 in endothelial cells remain unclear.

Direct targeting of VEGF and its receptor (VEGFR) by miR-150 is found in colorectal cancer [76]. Accordingly, the down-regulation of miR-150 in colorectal cancer has a negative correlation with angiogenesis induction.

## Conclusion

MiR-150 has an ectopic expression during solid tumor metastasis and it is involved in EMT, migration, invasion, and angiogenesis. Reviewing the various studies on the role of miR-150 in EMT, cell proliferation, apoptosis, and angiogenesis processes revealed that miR-150 is up or down-regulated during tumor progression and targets a wide range of oncogene or tumor suppressor genes. Transcription factors such as ZEB1, HMGA2, FOXO4 and c-Myb, and key signaling pathways including Wnt/β-catenin and TGFβ are determined as miR-150 targets in the EMT process. Furthermore, miR-150 regulates cancer cell migration by affecting multiple effectors including matrix metalloproteinases (MMP14 and MMP13), cell adhesion molecules (ITGA3, ITGA6), transcription factors (MYB), and epigenetics factors (HMGA2 and EZH2). By interacting with cell surface receptors and lncRNAs, miR-150 exerts its regulatory role on cancer cell proliferation and apoptosis. The expression of miR-150 is frequently reduced in tumor cell migration and serves as a tumor suppressor. However, the level of miR-150 in the migration process of non-cancerous cells can increase or decrease. Concerning these findings, miR-150 levels are modulated by upstream signaling pathways, tumor-associated cells, and lncRNAs, which can vary based on cancer cell type, gene expression profile, tumor microenvironment, and pathological circumstances. Furthermore, the target genes of miR-150 determine its function as a tumor suppressor miRNA or oncomiR, and EMT, migration, cell proliferation, apoptosis, and angiogenesis are inhibited or induced. To completely understand the processes by which miR-150 influences metastasis in various cancer types and to investigate its potential as a therapeutic target for cancer therapy, further study is required.

## Data Availability

Not applicable.

## References

[1] Chambers AF, Groom AC, I.C.J.N.R C, MacDonald (2002). Dissemination and Growth of cancer Cells in Metastatic Sites.

[2] Pouysségur J, Dayan F, Mazure NMJN (2006). Hypoxia Signal cancer Approaches Enforce Tumour Regres.

[3] Weinberg RA, Weinberg RA. The biology of cancer. WW Norton & Company; 2006.

[4] Chiang AC, Massagué J (2008). Molecular basis of Metastasis. N Engl J Med.

[5] Lee RC, Feinbaum RL, Ambros V (1993). The C. Elegans heterochronic gene lin-4 encodes small RNAs with antisense complementarity to lin-14. Cell.

[6] Plotnikova O, Baranova A. and M.J.F.i.g. Skoblov, *Comprehensive analysis of human microRNA–mRNA interactome* 2019: p. 933. 10.3389/fgene.2019.00933 PMC6792129 31649721

[7] Landthaler M, Yalcin A, Tuschl T (2004). The human DiGeorge syndrome critical region gene 8 and its D. Melanogaster homolog are required for miRNA biogenesis. Curr Biol.

[8] Kim TH (2017). miR-150 enhances apoptotic and anti-tumor effects of paclitaxel in paclitaxel-resistant Ovarian cancer cells by targeting Notch3. Oncotarget.

[9] Sun W (2016). MicroRNA-150 suppresses cell proliferation and Metastasis in hepatocellular carcinoma by inhibiting the GAB1-ERK axis. Oncotarget.

[10] Yang K (2015). A decrease in miR-150 regulates the malignancy of Pancreatic cancer by targeting c-Myb and MUC4. Pancreas.

[11] Koshizuka K (2017). Deep sequencing-based microRNA expression signatures in head and neck squamous cell carcinoma: dual strands of pre-mir-150 as antitumor miRNAs. Oncotarget.

[12] Huang S (2013). miR-150 promotes human Breast cancer growth and malignant behavior by targeting the pro-apoptotic purinergic P2 × 7 receptor. PLoS ONE.

[13] Cascione L (2013). Integrated microRNA and mRNA signatures associated with survival in triple negative Breast cancer. PLoS ONE.

[14] Yang J (2020). Guidelines and definitions for research on epithelial–mesenchymal transition. Nat Rev Mol Cell Biol.

[15] Ansieau S, Collin G, Hill L (2014). EMT or EMT-Promoting transcription factors, where to focus the light?. Front Oncol.

[16] Alidadiani N (2018). Epithelial mesenchymal transition transcription factor (TF): the structure, function and microRNA feedback loop. Gene.

[17] Hartwig A (2001). Zinc finger proteins as potential targets for toxic metal ions: differential effects on structure and function. Antioxid Redox Signal.

[18] Jin MF et al. *MicroRNA-150 predicts a favorable prognosis in patients with epithelial Ovarian Cancer, and inhibits Cell Invasion and Metastasis by suppressing Transcriptional Repressor ZEB1*. PLoS ONE, 2014. 9(8). 10.1371/journal.pone.0103965 PMC4121232 25090005

[19] Yokobori T (2013). MiR-150 is associated with poor prognosis in esophageal squamous cell carcinoma via targeting the EMT inducer ZEB1. Cancer Sci.

[20] Sánchez-Tilló E (2010). ZEB1 represses E-cadherin and induces an EMT by recruiting the SWI/SNF chromatin-remodeling protein BRG1. Oncogene.

[21] Lu W et al. *Long non-coding RNA linc00673 regulated non-small cell Lung cancer proliferation, migration, invasion and epithelial mesenchymal transition by sponging miR-150-5p*. Mol Cancer, 2017. 16. 10.1186/s12943-017-0685-9 PMC5504775 28697764

[22] Jin H (2019). Long non-coding RNA MIAT competitively binds mir-150-5p to regulate ZEB1 expression in osteosarcoma. Oncol Lett.

[23] López-Urrutia E (2019). Crosstalk between long non-coding RNAs, Micro-RNAs and mRNAs: deciphering Molecular mechanisms of Master regulators in Cancer. Front Oncol.

[24] Liu DK (2021). MiR-150 suppressed cell viability, invasion and EMT via HMGA2 in oral squamous cell carcinoma. Eur Rev Med Pharmacol Sci.

[25] Watanabe S (2009). HMGA2 maintains oncogenic RAS-induced epithelial-mesenchymal transition in human Pancreatic cancer cells. Am J Pathol.

[26] Li H et al. *MiR-150 promotes cellular Metastasis in non-small cell Lung cancer by targeting FOXO4*. Sci Rep, 2016. 6. 10.1038/srep39001 PMC5157020 27976702

[27] Eijkelenboom A, Burgering BMT (2013). FOXOs: signalling integrators for homeostasis maintenance. Nat Rev Mol Cell Biol.

[28] Xu MM (2014). Low expression of the FoxO4 gene may contribute to the phenomenon of EMT in non-small cell Lung cancer. Asian Pac J Cancer Prev.

[29] Tung CH (2020). MicroRNA-150-5p promotes cell motility by inhibiting c-Myb-mediated slug suppression and is a prognostic biomarker for recurrent Ovarian cancer. Oncogene.

[30] Tanno B (2010). Expression of slug is regulated by c-Myb and is required for invasion and bone marrow homing of cancer cells of different origin. J Biol Chem.

[31] Knopfová L (2012). c-Myb regulates matrix metalloproteinases 1/9, and cathepsin D: implications for matrix-dependent Breast cancer cell invasion and Metastasis. Mol Cancer.

[32] Debnath P et al. *Epithelial-mesenchymal transition and its transcription factors*. Biosci Rep, 2022. 42(1). 10.1042/BSR20211754 PMC8703024 34708244

[33] Guo YH (2016). Wnt/beta-catenin pathway transactivates microRNA-150 that promotes EMT of Colorectal cancer cells by suppressing CREB signaling. Oncotarget.

[34] Sakamoto KM, Frank DA (2009). CREB in the pathophysiology of cancer: implications for targeting transcription factors for cancer therapy. Clin Cancer Res.

[35] Zimmerman NP (2015). Cyclic AMP regulates the migration and invasion potential of human Pancreatic cancer cells. Mol Carcinog.

[36] Ionov Y, Matsui S, Cowell JK (2004). A role for p300/CREB binding protein genes in promoting cancer progression in colon cancer cell lines with microsatellite instability. Proc Natl Acad Sci U S A.

[37] Hong X, Yu JJ (2019). MicroRNA-150 suppresses epithelial-mesenchymal transition, invasion, an Metastasis in Prostate cancer through the TRPM4-mediated beta-catenin signaling pathway. Am J Physiology-Cell Physiol.

[38] Demion M et al. *Trpm4 gene invalidation leads to cardiac hypertrophy and electrophysiological alterations*. 2014. 9(12): p. e115256. 10.1371/journal.pone.0115256 PMC4274076 25531103

[39] Scher HI (2012). Increased survival with enzalutamide in Prostate cancer after chemotherapy. N Engl J Med.

[40] Zhu JM, Han SC (2019). Mir-150-5p promotes the proliferation and epithelial-mesenchymal transition of cervical carcinoma cells via targeting SRCIN1. Pathol Res Pract.

[41] Ortiz MA (2021). Src family kinases, adaptor proteins and the actin cytoskeleton in epithelial-to-mesenchymal transition. Cell Communication and Signaling.

[42] Cao M (2014). miR-150 promotes the proliferation and migration of Lung cancer cells by targeting SRC kinase signalling inhibitor 1. Eur J Cancer.

[43] Quan XY (2019). MicroRNA-150-5p and SRC kinase signaling inhibitor 1 involvement in the pathological development of gastric cancer. Experimental and Therapeutic Medicine.

[44] Lu QF, Guo ZJ, Qian HX (2019). Role of microRNA-150-5p/SRCIN1 axis in the progression of Breast cancer. Experimental and Therapeutic Medicine.

[45] Sun SC (2017). The non-canonical NF-kappaB pathway in immunity and inflammation. Nat Rev Immunol.

[46] Nishida K, Hirano T (2003). The role of Gab family scaffolding adapter proteins in the signal transduction of cytokine and growth factor receptors. Cancer Sci.

[47] Shin S et al. *ERK2 regulates epithelial-to-mesenchymal plasticity through DOCK10-dependent Rac1/FoxO1 activation*. 2019. 116(8): p. 2967–76. 10.1073/pnas.1811923116 PMC6386703 30728292

[48] Massagué J (2012). TGFβ signalling in context. Nat Rev Mol Cell Biol.

[49] Syed V (2016). TGF-β signaling in Cancer. J Cell Biochem.

[50] Zhou HF (2021). Knockdown of circular RNA VANGL1 inhibits TGF-beta-induced epithelial-mesenchymal transition in Melanoma cells by sponging miR-150-5. J Cell Mol Med.

[51] Jeck WR. And N.E.J.N.b. Sharpless, *Detecting and characterizing circular RNAs*. 2014. 32(5): p. 453–61. 10.1038/nbt.2890 PMC4121655 24811520

[52] Yamaguchi H, Wyckoff J, Condeelis J (2005). Cell migration in tumors. Curr Opin Cell Biol.

[53] Ladoux B, Mège RM, Trepat X (2016). Front-rear polarization by mechanical cues: from single cells to tissues. Trends Cell Biol.

[54] Wang W (2005). Tumor cells caught in the act of invading: their strategy for enhanced cell motility. Trends Cell Biol.

[55] Winkler J (2020). Concepts of extracellular matrix remodelling in tumour progression and Metastasis. Nat Commun.

[56] Piccinini AM, Midwood KS (2014). Illustrating the interplay between the extracellular matrix and microRNAs. Int J Exp Pathol.

[57] Zhang Z, et al. MicroRNA-150 promotes cell proliferation, migration, and invasion of Cervical cancer through targeting PDCD4. Volume 97. Biomedicine & Pharmacotherapy; 2018. pp. 511–7. 10.1016/j.biopha.2017.09.143 29091902

[58] Sakr M (2016). Mir-150-5p and miR-133a suppress glioma cell proliferation and migration through targeting membrane-type-1 matrix metalloproteinase. Gene.

[59] Knapinska AM, Fields GB. *The expanding role of MT1-MMP in Cancer Progression*. Pharmaceuticals (Basel), 2019. 12(2). 10.3390/ph12020077 PMC6630478 31137480

[60] Li T (2014). Mir-150-5p inhibits hepatoma cell migration and invasion by targeting MMP14. PLoS ONE.

[61] Xiao GD (2019). FAM83A-AS1 promotes lung adenocarcinoma cell migration and invasion by targeting mir-150-5p and modifying MMP14. Cell Cycle.

[62] Shi GH, et al. Long non-coding RNA LINC00511/miR-150/MMP13 axis promotes Breast cancer proliferation, migration and invasion. Volume 1867. Biochimica Et Biophysica Acta-Molecular Basis of Disease; 2021. 3. 10.1016/j.bbadis.2020.165957 33031905

[63] Okato A (2017). Dual strands of pre-mir-150 (miR-150-5p and miR-150-3p) act as antitumor miRNAs targeting SPOCK1 in naive and castration-resistant Prostate cancer. Int J Oncol.

[64] Koshizuka K (2018). Antitumor Mir-150-5p and mir-150-3p inhibit cancer cell aggressiveness by targeting SPOCK1 in head and neck squamous cell carcinoma. Auris Nasus Larynx.

[65] Alshargabi R (2020). SPOCK1 is a novel inducer of epithelial to mesenchymal transition in drug-induced gingival overgrowth. Sci Rep.

[66] Gregory RI (2005). Human RISC couples microRNA biogenesis and posttranscriptional gene silencing. Cell.

[67] Chendrimada TP (2005). TRBP recruits the Dicer complex to Ago2 for microRNA processing and gene silencing. Nature.

[68] Silvers CR (2021). Tenascin-C expression in the lymph node pre-metastatic niche in muscle-invasive Bladder cancer. Br J Cancer.

[69] Takada Y, Ye X, Simon S (2007). The integrins. Genome Biol.

[70] Wang WH (2014). MiR-150-5p suppresses Colorectal cancer cell migration and invasion through targeting MUC4. Asian Pac J Cancer Prev.

[71] Srivastava SK (2011). MicroRNA-150 directly targets MUC4 and suppresses growth and malignant behavior of Pancreatic cancer cells. Carcinogenesis.

[72] Zhang Y (2022). LINC00857 regulated by ZNF460 enhances the expression of CLDN12 by sponging mir-150-5p and recruiting SRSF1 for alternative splicing to promote epithelial-mesenchymal transformation of pancreatic adenocarcinoma cells. RNA Biol.

[73] Qin G (2020). MicroRNA and transcription factor co-regulatory networks and subtype classification of seminoma and non-seminoma in testicular germ cell tumors. Sci Rep.

[74] Mullany LE (2018). MicroRNA-transcription factor interactions and their combined effect on target gene expression in colon Cancer cases. Genes Chromosomes Cancer.

[75] Su XY (2019). miR-150 suppresses Tumor Growth in Melanoma through downregulation of MYB. Oncol Res.

[76] Li C (2018). MicroRNA-150 inhibits the proliferation and Metastasis potential of Colorectal cancer cells by targeting iASPP. Oncol Rep.

[77] He S et al. *The Tumor suppressor p53 can promote collective cellular migration*. 2019. 14(2): p. e0202065. 10.1371/journal.pone.0202065 PMC6358060 30707705

[78] Thorenoor N (2016). Long non-coding RNA ZFAS1 interacts with CDK1 and is involved in p53-dependent cell cycle control and apoptosis in Colorectal cancer. Oncotarget.

[79] Li T (2015). Amplification of long noncoding RNA ZFAS1 promotes Metastasis in Hepatocellular Carcinoma. Cancer Res.

[80] Nie F (2017). Long noncoding RNA ZFAS1 promotes gastric cancer cells proliferation by epigenetically repressing KLF2 and NKD2 expression. Oncotarget.

[81] Xia B (2017). Long non-coding RNA ZFAS1 interacts with mir-150-5p to regulate Sp1 expression and Ovarian cancer cell malignancy. Oncotarget.

[82] Zhou SY (2020). lncRNA MIAT regulates Cell Growth, Migration, and Invasion through sponging mir-150-5p in Ovarian Cancer. Cancer Biotherapy and Radiopharmaceuticals.

[83] Tao BB (2017). Evidence for the association of chromatin and microRNA regulation in the human genome. Oncotarget.

[84] Tang WT (2018). MicroRNA-150 suppresses triple-negative Breast cancer Metastasis through targeting HMGA2. Oncotargets and Therapy.

[85] Cai ZL (2020). The pro-migration and anti-apoptosis effects of HMGA2 in HUVECs stimulated by hypoxia. Cell Cycle.

[86] Guo K et al. *LncRNA-MIAT promotes thyroid cancer progression and function as ceRNA to target EZH2 by sponging miR-150-5p* Cell Death & Disease, 2021. 12(12). 10.1038/s41419-021-04386-0 PMC8608816 34811354

[87] Chien YC (2018). EZH2 promotes migration and invasion of triple-negative Breast cancer cells via regulating TIMP2-MMP-2/-9 pathway. Am J Cancer Res.

[88] Chang CJ, Hung MC (2012). The role of EZH2 in tumour progression. Br J Cancer.

[89] The Lancet H (2020). Proliferation and differentiation. Lancet Haematol.

[90] Houston JP, Cytometry A. 2019. 95(6): p. 655–656. 10.1002/cyto.a.23837 31207048

[91] Farhana L (2013). Upregulation of miR-150* and miR-630 induces apoptosis in Pancreatic cancer cells by targeting IGF-1R. PLoS ONE.

[92] Rotondo JC et al. *The role of Purinergic P2X7 receptor in inflammation and Cancer: Novel Molecular insights and clinical applications*. Cancers (Basel), 2022. 14(5). 10.3390/cancers14051116 PMC8909580 35267424

[93] Wang X (2020). LncRNA NEAT1 regulates 5-Fu sensitivity, apoptosis and Invasion in Colorectal Cancer through the MiR-150-5p/CPSF4 Axis. Onco Targets Ther.

[94] Song Y (2022). CPSF4 promotes tumor-initiating phenotype by enhancing VEGF/NRP2/TAZ signaling in Lung cancer. Med Oncol.

[95] Zhang M et al. *Overproduced CPSF4 Promotes Cell Proliferation and Invasion via PI3K-AKT Signaling Pathway in Oral Squamous Cell Carcinoma* J Oral Maxillofac Surg, 2021. 79(5): p. 1177.e1-1177.e14. 10.1016/j.joms.2020.12.047 33535057

[96] Lee K (2021). CPSF4 promotes triple negative Breast cancer Metastasis by upregulating MDM4. Signal Transduct Target Ther.

[97] Chen W (2014). CPSF4 activates telomerase reverse transcriptase and predicts poor prognosis in human lung adenocarcinomas. Mol Oncol.

[98] Li B (2022). Repression of lncRNA PART1 attenuates Ovarian cancer cell viability, migration and invasion through the miR-503-5p/FOXK1 axis. BMC Cancer.

[99] Ran R (2022). Long non–coding RNA PART1: dual role in cancer. Hum Cell.

[100] Zhou T (2020). LncRNA PART1 regulates Colorectal cancer via targeting miR-150-5p/miR-520 h/CTNNB1 and activating Wnt/β-catenin pathway. Int J Biochem Cell Biol.

[101] Ledinek M, Sobočan, Knez J. *The Role of CTNNB1 in Endometrial Cancer* Dis Markers, 2022. 2022: p. 1442441. 10.1155/2022/1442441 PMC9072012 35531470

[102] Jia H (2021). Regulatory effect of the MAFG–AS1/miR–150–5p/MYB axis on the proliferation and migration of Breast cancer cells. Int J Oncol.

[103] Cicirò Y, Sala A (2021). MYB oncoproteins: emerging players and potential therapeutic targets in human cancer. Oncogenesis.

[104] Nishida N (2006). Angiogenesis in cancer. Vasc Health Risk Manag.

[105] Rajabi M, Mousa SA. *The role of Angiogenesis in Cancer Treatment*. Biomedicines, 2017. 5(2). 10.3390/biomedicines5020034 PMC5489820 28635679

[106] Landskroner-Eiger S, Moneke I, Sessa WC (2013). miRNAs as modulators of angiogenesis. Cold Spring Harb Perspect Med.

[107] Liu YC (2013). Microvesicle-delivery miR-150 promotes tumorigenesis by up-regulating VEGF, and the neutralization of miR-150 attenuate Tumor development. Protein Cell.

[108] Colla S (2007). The new tumor-suppressor gene inhibitor of growth family member 4 (ING4) regulates the production of proangiogenic molecules by Myeloma cells and suppresses hypoxia-inducible factor-1 alpha (HIF-1alpha) activity: involvement in myeloma-induced angiogenesis. Blood.

[109] Li J (2013). Microvesicle-mediated transfer of MicroRNA-150 from monocytes to endothelial cells promotes angiogenesis. J Biol Chem.

[110] Wang WB (2014). MiR-150 enhances the motility of EPCs in vitro and promotes EPCs homing and thrombus resolving in vivo. Thromb Res.

[111] Perales G (2022). MicroRNA-150-5p is upregulated in the brain microvasculature during prenatal alcohol exposure and inhibits the angiogenic factor Vezf1. Alcoholism-Clinical and Experimental Research.

[112] Bian Y (2021). Mir-150-5p affects AS plaque with ASMC proliferation and migration by STAT1. Open Med.

[113] Zeng Y (2020). Mir-150-5p mediates extravillous trophoblast cell migration and angiogenesis functions by regulating VEGF and MMP9. Placenta.

[114] Liu Z (2015). MicroRNA-150 protects the heart from injury by inhibiting monocyte accumulation in a mouse model of acute Myocardial Infarction. Circ Cardiovasc Genet.

[115] Chen M (2017). MicroRNA-150 attenuates hypoxia-induced excessive proliferation and migration of pulmonary arterial smooth muscle cells through reducing HIF-1α expression. Biomed Pharmacother.

[116] He QW (2016). MiR-150 regulates Poststroke Cerebral Angiogenesis via Vascular endothelial growth factor in rats. CNS Neurosci Ther.

[117] Chen ML (2023). Knockdown of mir-150-5p reduces hypoxia-induced autophagy and epithelial-mesenchymal transition of endometriotic cells via regulating the PDCD4/NF-κB signaling pathway. Cytokine.

[118] Tano N, Kim HW, Ashraf M (2011). microRNA-150 regulates mobilization and migration of bone marrow-derived mononuclear cells by targeting Cxcr4. PLoS ONE.

[119] Liu CH (2015). Endothelial microRNA-150 is an intrinsic suppressor of pathologic ocular neovascularization. Proc Natl Acad Sci U S A.

[120] Lee JH et al. *MiR-150-5p May Contribute to Pathogenesis of Human Leiomyoma via Regulation of the Akt/p27(Kip1) pathway in Vitro*. Int J Mol Sci, 2019. 20(11). 10.3390/ijms20112684 PMC6601023 31159158

